# Spurious Resonance of the QCM Sensor: Load Analysis Based on Impedance Spectroscopy

**DOI:** 10.3390/s23104939

**Published:** 2023-05-21

**Authors:** Ioan Burda

**Affiliations:** Physics Department, Babes-Bolyai University, 400084 Cluj-Napoca, Romania; ioan.burda@ubbcluj.ro

**Keywords:** QCM sensor, impedance spectroscopy, spurious resonances, virtual impedance analyzer, AT-cut quartz crystal, extended BVD electrical model

## Abstract

A research topic of equal importance to technological and application fields related to quartz crystal is the presence of unwanted responses known as spurious resonances. Spurious resonances are influenced by the surface finish of the quartz crystal, its diameter and thickness, and the mounting technique. In this paper, spurious resonances associated with fundamental resonance are studied by impedance spectroscopy to determine their evolution under load conditions. Investigation of the response of these spurious resonances provides new insights into the dissipation process at the QCM sensor surface. The significant increase of the motional resistance for spurious resonances at the transition from air to pure water is a specific situation revealed experimentally in this study. It has been shown experimentally that in the range between the air and water media, spurious resonances are much more attenuated than the fundamental resonance, thus providing support for investigating the dissipation process in detail. In this range, there are many applications in the field of chemical sensors or biosensors, such as VOC sensors, humidity sensors, or dew point sensors. The evolution of D factor with increasing medium viscosity is significantly different for spurious resonances compared to fundamental resonance, suggesting the usefulness of monitoring them in liquid media.

## 1. Introduction

Quartz crystal is a natural substance that exhibits a piezoelectric effect and is chemically and mechanically very stable. Its properties have been of great interest since the early days of electronics. Quartz crystal performs electrically like a series circuit consisting of resistance, inductance, and capacitance (RLC). Unlike other materializations of the RLC circuit, quartz crystal has very low attenuation at series resonance [1]. Chemically, quartz crystal is silicon dioxide SiO_2_ with no naturally occurring quality suitable for the production of electronic devices. For this reason, technologies for the production of high-purity synthetic quartz crystals have been successfully developed [2,3]. The quality of synthetic quartz crystal is a function of the growth rate, which is about 1 mm/day. A slow growth rate results in a more homogeneous quartz crystal with fewer impurities incorporated into the crystal lattice [4]. A high-performance version of the quartz crystal is SC-cut, a double-rotated cut that minimizes resonant frequency changes due to temperature gradients. SC-cut quartz crystals have an inflection point at around 92 °C. In addition to the inflection point at high temperatures, the temperature dependence is described by a smooth cubic relationship, and they are therefore less affected by temperature deviations from the inflection point. Due to its outstanding qualities, the SC-cut quartz crystal version [5] has become a promising candidate for detecting high-frequency gravitational waves [6].

An important step in the spread of the use of quartz crystal as an electronic device in various applications was made by the generation of its AT-cut version [7,8,9]. AT-cut quartz crystals are singularly rotated cuts on the Y-axis in which the upper and lower halves of the crystal move in opposite directions, causing a thickness-shear vibration during mechanical oscillation. The relationship between temperature and resonant frequency of the quartz crystal is cubic, with an inflection point near room temperature [10]. Consequently, the use of AT-cut quartz crystal is effective at or near room temperature, ~25 °C. The standard electronic device is a quartz crystal disc with metal electrodes deposited on both sides by evaporation. An alternating electric field is applied to these electrodes, which stimulates the thickness-shear oscillation of the quartz crystal. In the case of AT-cut quartz crystal, only odd harmonics are possible, because in the case of even harmonics, the electrodes have the same polarity, and no electric field is present to stimulate the mechanical oscillation. The odd harmonic oscillations are the third, fifth, seventh, and ninth; they are further called overtones with the caveat that the upper harmonic oscillation is not exactly an integer multiple of the fundamental frequency of the quartz crystal.

Quartz crystal microbalance (QCM) has a successful application in the field of label-free sensors known as a QCM sensor [11,12,13,14]. The electrical properties of the QCM sensor as a function of frequency can be described in the vicinity of the resonance frequency by an equivalent circuit known as the Butterworth-Van Dyke (BVD) model [15,16]. In the BVD model, the oscillating mass is symbolized by a motional inductance, while the elasticity is represented by a motional capacitance. Frequently in QCM sensor applications, it is important to measure mass changes on its surface, and for this purpose only, the change in the series resonance frequency of the fundamental oscillation mode is considered. 

In the case of advanced QCM methods [17,18], changes in the series resonance frequency as well as the dissipative process induced by interactions occurring on the electrode surface are tracked. The dissipative process is expressed by at least one of the following parameters: bandwidth, quality factor (Q-factor), dissipation factor (D-factor), or motional resistance from the BVD model. Expressed in different forms, the above parameters quantify the processes that extract energy from the QCM sensor during oscillation. More information can be obtained by determining all the electrical parameters of the BVD model [19] and even more by tracking the changes of the electrical parameters for the overtones of the series fundamental resonant frequency [20,21]. The different response of the overtones compared to the fundamental series resonance in the case of a viscoelastic film ensures that more information is extracted from the collected QCM data, providing a better understanding of what is happening at the sensor surface. Extracting new information by extending the fundamental resonance and overtone resonance monitoring of the QCM sensor has been and continues to be a constant concern, which, over time, has led to its current success.

A research topic of equal importance to technological and application fields related to quartz crystal was and is generated by the presence of unwanted responses known as spurious resonances [22,23]. Quartz crystal resonators produce, for fundamental resonance and each overtone, a principal mode, which is a bulk shear vibration, as well as spurious responses, which are anharmonic modes, typically thickness-shear modes greater than the resonant frequency. Spurious resonances are influenced by the surface finish of the quartz crystal, its diameter and thickness, and the mounting technique. The spurious resonances disrupt the ring-down measurement method [18,24] by causing a frequency and amplitude modulation of the exponentially damped sine wave.

In this paper, spurious resonances associated with fundamental resonance are studied by impedance spectroscopy in order to determine their evolution under load conditions. Investigating the response of these spurious resonances provides insight into the dissipation process in the viscoelastic film involved in the functionalization. The original contributions of the paper are (i) adapting a virtual impedance analyzer (VIA) to a large scanning range and calculating the parameters of the extended BVD model, (ii) investigating the response of the QCM sensor for spurious resonances under load conditions, and (iii) demonstrating the usefulness of measuring and tracking the electrical parameters of the extended BVD model for spurious resonances of the QCM sensor. In the remainder of the paper, Section 2 presents the extended BVD electrical model, some theoretical concepts, and VIA; Section 3 presents the experimental results of impedance spectroscopy and the evolution of key electrical parameters for spurious resonances; and Section 4 and Section 5 are devoted to the discussion and conclusions.

## 2. Materials and Methods

### 2.1. Extended BVD Model of the QCM Sensor

**Figure 1:**
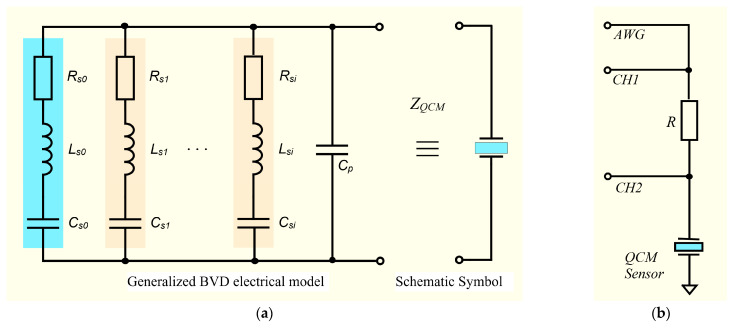
(**a**) Extended BVD electric model of the QCM sensor, (**b**) half-bridge configuration for the virtual impedance analyzer.

The BVD model is used to describe the behavior of the QCM sensor as a function of frequency, and its circuit can have multiple motional branches to account for multiple resonances or vibration modes, as shown in Figure 1a. Through this extension of the standard BVD model circuit, the electrical properties of the QCM sensor can be modeled in the vicinity of several resonance frequencies.

Measurement of all electrical parameters of the extended BVD model is possible using a virtual impedance analyzer with a large scanning range. The VIA front-end hardware component is shown in Figure 1b. As can be seen, this is a simple half-bridge configuration that permits simultaneous electrochemical measurements due to the grounding of the electrode exposed to the operating medium.

Simulation via Matlab^®^ scripts is limited to Bode and Nyquist plot generation, which can provide maximum information about the complex behavior of the QCM sensor. To generate Bode and Nyquist plots for the extended BVD model in Figure 1b, the impedance of the motional branches formed from series RLC circuits in the resonant frequencies range of the QCM sensor is calculated using the following equation:

(1) $$Z_{si}\left(j\omega \right)=R_{si}+j\omega L_{si}+\frac{1}{j\omega C_{si}},\;\;for\;\;i=0,\;1,\;2,\;\ldots$$

The branch formed by the parallel capacitance has the impedance of the following form:

(2) $$Z_{p}\left(j\omega \right)=\frac{1}{j\omega C_{p}}$$

Considering the extended BVD model for spurious resonances, the QCM sensor impedance is calculated using the following equation:

(3) $$Z_{QCM}\left(j\omega \right)=Z_{so}\left(j\omega \right)\left|\left|Z_{s1}\left(j\omega \right)\right|\right|Z_{s2}\left(j\omega \right)||Z_{s3}\left(j\omega \right),\;\ldots ,\;||Z_{p}\left(j\omega \right)$$

The spurious resonances can arise from various sources, such as mechanical stresses, acoustic wave reflections, and parasitic capacitances and inductances. 

### 2.2. Simulation of the Extended BVD Model of the QCM Sensor

Investigation of spurious resonances is not frequently encountered in the literature [22], or, in other words, it is limited to reporting their presence [23]. Spurious resonances usually occur at frequencies higher than the fundamental frequency and immediately afterwards and can affect the accuracy of measurements. By considering monitoring spurious resonances of the QCM sensor, it is possible to accurately measure the mass and viscoelastic properties of thin films. 

Considering the QCM sensor in the air, some values of the electrical parameters for the simulation of the extended BVD model are proposed in Table 1. These electrical parameter values were suggested by experimental investigations during the development of the extended scanning range VIA.

**Figure 2:**
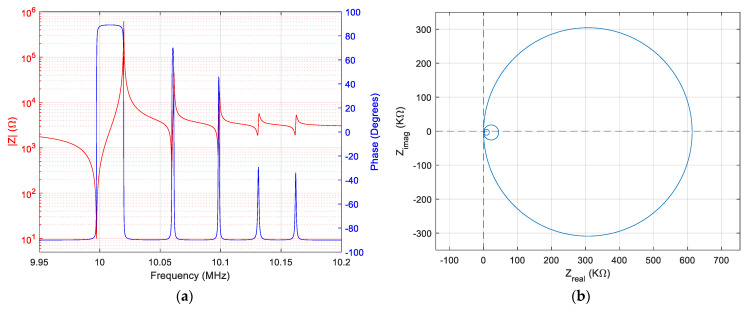
Simulation of the extended BVD model of the QCM sensor in air: (**a**) Bode plot, (**b**) Nyquist plot.

For the values of the electrical parameters of the extended BVD model in Table 1, Bode and Nyquist plots are engendered in Matlab^®^ and shown in Figure 2.

In the simulated case, fundamental resonance is dominant, and this simulated situation is frequently encountered experimentally and is also illustrated in the Nyquist diagram in Figure 2b. This relatively ideal situation simulated in Figure 1 is not found in all cases. In many cases, depending on quartz crystal manufacturing technology and destination, their response may differ substantially.

In the various applications of quartz crystals, such as oscillators, the ratio of the series resonance motional resistance $R_{s0}$ to spurious series resonance motional resistance $R_{si}$ is important and is, by definition, expressed by the following equation:

(4) $$A=20\log\left(\frac{R_{s0}}{R_{si}}\right)$$

Normally, values between 3 dB and 6 dB may be sufficient for general purpose frequency reference applications. For quartz crystal filters, attenuations higher than 40 dB are often required to avoid distorting their response. This performance can only be achieved with special design techniques and involves the use of very small values of motional capacitance. 

Simulating the response of the QCM sensor in a liquid medium is extremely important from the perspective of its use as a biosensor. Table 2 shows the values of the electrical parameters of the extended BVD model proposed for simulation. In this case, only one of the QCM sensor armatures is considered to be in contact with water. Again, values as close as possible to the experimental values were chosen.

**Figure 3:**
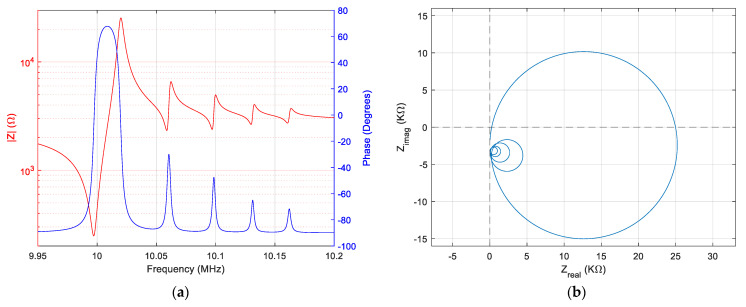
Simulation of the extended BVD model of the QCM sensor in water: (**a**) Bode plot, (**b**) Nyquist plot.

The result of the Matlab^®^ simulation in this case is shown in Figure 3a, the value of the motional resistances being strongly modified. Similarly, in Figure 3b, the same profound change in ratios can be seen in this case in the Nyquist plot.

By understanding and controlling spurious resonances, the accuracy and precision of QCM measurements can be improved, allowing more reliable analysis and interpretation of the data. This can be particularly important in applications such as volatile organic compound (VOC) monitoring, where the QCM sensor is used to detect and quantify interactions in the functional layer. In addition, the study of spurious resonances can provide valuable information about the mechanical and environmental factors affecting QCM sensor performance. This knowledge can be used to optimize the design and operation of QCM sensors, leading to improved sensitivity and accuracy in various applications. In addition, the study of QCM sensor spurious resonances can lead to the development of new, more accurate, and reliable detection strategies and technologies.

### 2.3. Virtual Impedance Analyzer and Expermental Setup

**Figure 4:**
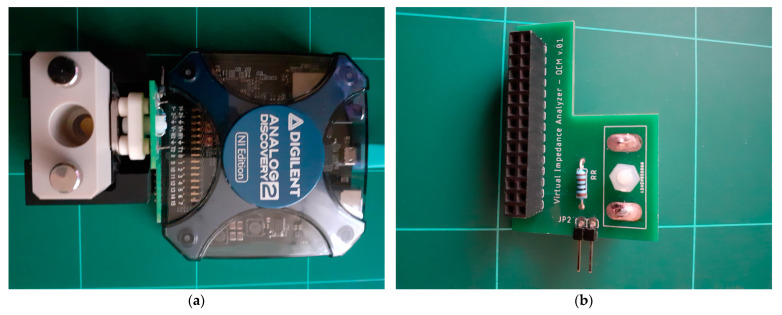
Wide scanning range virtual impedance analyzer: (**a**) experimental setup, (**b**) half-bridge shield.

For the experimental investigations in this paper, a VIA built around the Analog Discovery 2 (AD2) virtual instrument from Digilent Inc, Pullman, WA, USA [19,25,26], was used. The AD2 virtual instrument provides the hardware and software support necessary for easy implementation of an impedance analyzer [27,28]. The experimental setup used to measure spurious resonances in various liquid media is shown in Figure 4a. The experimental setup is completed by the QCM flow cell kit (011121, ALS Co., Ltd., Tokyo, Japan) mounted in static measurement mode [29] to the shield built according to its size. The additional hardware component for the AD2 virtual instrument is based on the schematic in Figure 1b. The shield built for the impedance analyzer is shown in Figure 4b.

A Python module exploiting AD2 SDK (software development kit) functions provides acquisition and processing of raw experimental data. The method of finding spurious resonances is based on the search for minimum and maximum in impedance response, thus determining the frequency of series and parallel resonances for fundamental resonances and two spurious resonances. The value of the electrical parameters of the extended BVD model is based on their calculation from the raw experimental data of the QCM sensor impedance after setting the series resonance $\omega_{ri}$ and antiresonance $\omega_{ari}$ frequencies. The series resistor $R_{si}$ is implicitly determined in this case as a result of the minimum search function.

By measuring the impedance of the QCM sensor at a frequency $\omega_{m}$ about 10 times lower than the fundamental series resonance frequency (1 MHz), where its reactance $Z_{pm}=Z_{p}+Z_{stray}$ is purely capacitive, the shunt and stray capacitance are calculated based on the equation:

(5) $$C_{p}=\frac{1}{j\omega_{m}Z_{pm}}$$

It should be noted that the measurement of shunt capacitance and dispersion capacitance in a task precedes the measurement of the QCM sensor response. This allows automatic compensation of the QCM sensor to measure only the response for series branches. The following equation is used to achieve shunt and stray capacitance compensation in real time:

(6) $$Z_{s}\left(j\omega \right)=\frac{Z_{rm}Z_{pm}}{Z_{pm}-Z_{rm}}$$

where $Z_{rm}$ is the raw impedance measured at each point of the scan interval and is calculated from the following equation:

(7) $$Z_{rm}=R\left(\frac{V_{CH2}}{V_{CH1}-V_{CH2}}\right)=R\frac{V_{CH2}}{V_{R}}$$

The calculation of the parameters of the extended BVD model that cannot be determined directly from the raw experimental data is based on the following equations:

(8) $$C_{si}=C_{p}\left({\left(\frac{\omega_{ari}}{\omega_{ri}}\right)}^{2}-1\right)$$

(9) $$L_{si}=\frac{1}{\omega_{ri}^{2}C_{si}}$$

Other key parameters that better describe the energy dissipation process, such as the Q-factor or D-factor, can be calculated using the following equation:

(10) $$Q_{i}=\frac{1}{D_{i}}=\frac{1}{\omega_{ri}R_{si}C_{si}}=\frac{\omega_{ri}L_{si}}{R_{si}}$$

The Python module provides acquisition and processing of raw experimental data by calculating the electrical parameters of the extended BVD model for the fundamental resonance and the first two well-represented spurious resonances. The acquired raw experimental data are plotted together with the parameters of the extended BVD model as well as some key parameters specific to a QCM sensor.

## 3. Results

The experimental setup shown in Figure 4 contains a QCM sensor with a fundamental resonant frequency of 10 MHz (151225-10, International Crystal Manufacturing Co., Inc., Oklahoma City, OK, USA). The QCM sensor was clamped between the silicon O-rings of the static QCM cell with a minimum pressure following its response with VIA. During the measurements, the temperature in the laboratory was 21 ± 2 °C, with a relative humidity of 50 ± 10%. The VIA setup used for the acquisition of the raw experimental data was as follows: (i) passive excitation with a sinusoidal voltage with an amplitude of 1 V in the range of the series resonance and antiresonance frequencies of fundamental and spurious resonances and (ii) measurement of the QCM sensor impedance with a scan step of 1 Hz.

### 3.1. Load Analysis of the Spurious Resonances

As shown in the simulations presented above, a significant transformation of the frequency response of the QCM sensor occurs when the sensor changes from air to liquid medium (pure water). This QCM sensor transition covers commonly encountered situations with VOC sensors [30], humidity sensors [31], and dew point sensors [32], to name a few applications. Of particular interest from an application point of view are VOC sensors, especially in an array configuration, which is frequently encountered in an e-nose. Tracking the dissipation process in the case of the functionalized QCM sensor provides the experimental basis for an advanced understanding and description of the processes occurring at its surface. Spurious resonances may be useful, and from this hypothesis, the frequency response of the QCM sensor in various operating media is investigated experimentally.

**Figure 5:**
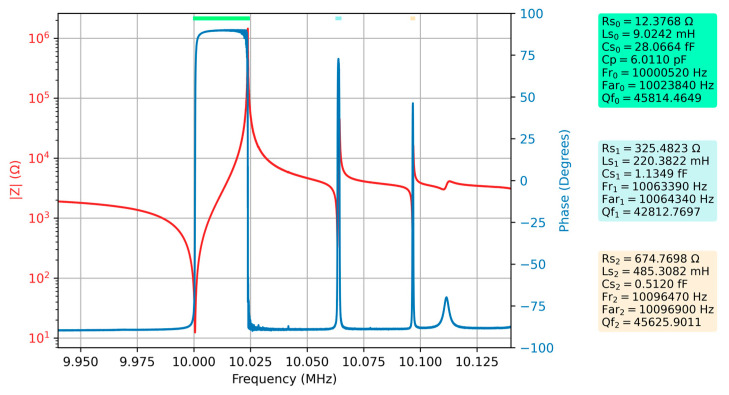
Bode plot of raw data for the QCM sensor in the air and the electrical parameters of the extended BVD model for the series resonance, respectively the first two spurious resonances.

Figure 5 shows the first measurement of the QCM sensor in air, still used as a reference. The electrical parameters of the extended BVD model as well as some other key parameters are automatically calculated by VIA and are also part of Figure 5. The fundamental resonance as well as spurious resonances are indicated in Figure 5 by a different color line at the top of the figure. The same color is used in the captions as background where the measured or calculated electrical parameters are shown.

**Figure 6:**
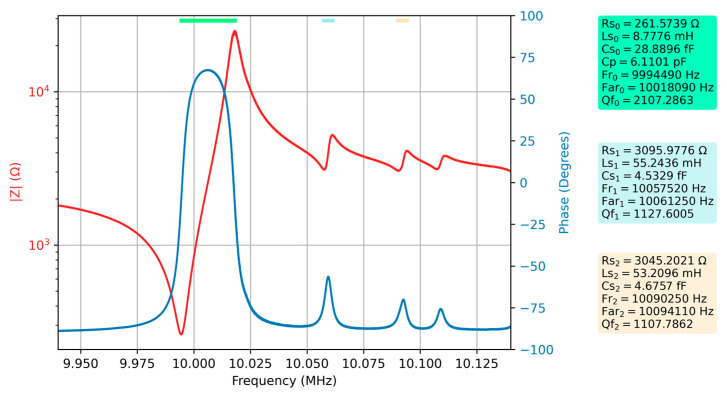
Bode plot of raw data for the QCM sensor in the water and the electrical parameters of the extended BVD model for the series resonance, respectively the first two spurious resonances.

After one of the QCM sensor armatures was covered with water, the measurement of the frequency response of the QCM sensor was repeated, and the results are shown in Figure 6 together with the new values of the electrical parameters.

A significant decrease in Q factor can be observed for the QCM sensor immersed with one of the armatures in the liquid medium. This significant attenuation also affects spurious resonances. To confirm the hypothesis that spurious resonances can provide useful information, a comparative study between the two measurements is considered below. Since the key parameters describing the energy dissipation process in the case of a QCM sensor depend, in turn, on the motional resistance according to Equation (10), only the evolution of this electrical parameter will be analyzed below. 

The values of the motional resistances In the two experimentally investigated cases are summarized in Table 3, as well as the differences between them. 

The change in motional resistance of the spurious resonances is much greater than the change in motional resistance for fundamental resonances. In other words, spurious resonances better highlight the dissipative processes taking place at the surface of the QCM sensor. Consequently, monitoring the motional resistances of spurious resonances during an experiment is a useful contribution from the perspective of increasing sensitivity to dissipation processes.

The evaluation of the effect of the increase in the viscosity of the liquid medium on the spurious resonances is justified under the hypothesis of confirming the usefulness of their monitoring in the case of the QCM sensor. It is also useful to identify the viscosity limit up to which an algorithm to identify the impedance local minimum and maximum specific to spurious resonances can be used. The effect of indeterminacy of the position of spurious resonances by the impedance minimum and maximum search algorithm, implemented in the Python module, was mainly avoided by a sequential search in subranges of the measurement range. 

**Figure 7:**
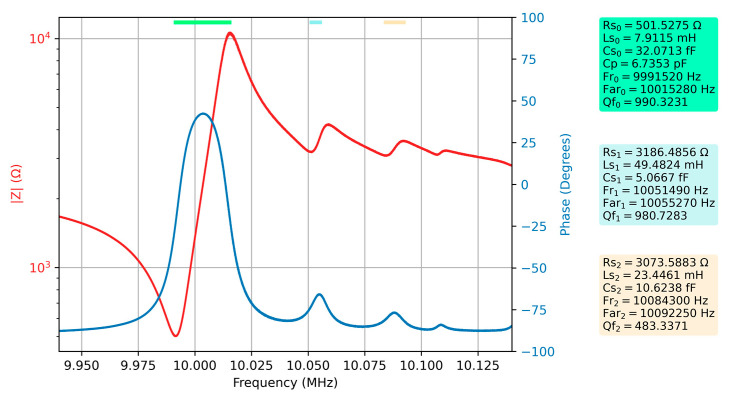
Bode plot of raw data for the QCM sensor in the 40% glycerin-water solution and the electrical parameters of the extended BVD model for the series resonance, respectively the first two spurious resonances.

The effect of increasing viscosity in liquid media on spurious resonances was investigated for glycerol-water solutions up to 80%, which is also their automatic identification limit for the implemented algorithm. Figure 7 shows the Bode plot together with the parameters of the extended BVD model for a 40% glycerin-water solution.

After a major change in the motional resistance for spurious resonances at the transition from air to water mentioned above, it can be seen that the increase in the viscosity of the liquid medium does not produce significant changes. In other words, the spurious resonances, although significantly attenuated at the transition to the liquid medium, show a robustness in relation to the increase in viscosity.

**Figure 8:**
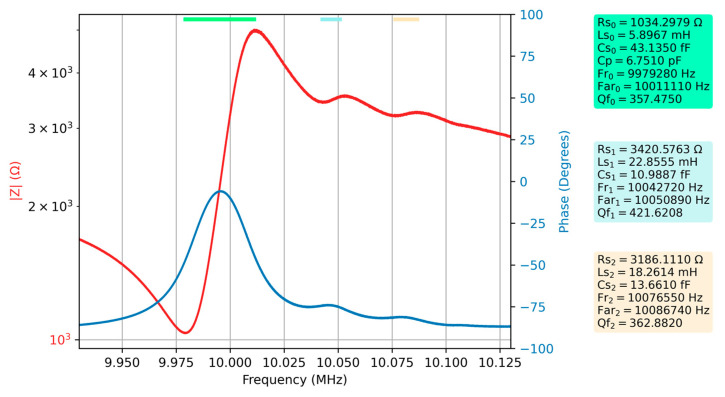
Bode plot of raw data for the QCM sensor in the 80% glycerin-water solution and the electrical parameters of the extended BVD model for the series resonance, respectively the first two spurious resonances.

Figure 8 shows the Bode plot for an 80% glycerin-water solution again following the evolution of the parameters of the extended BVD model.

As the viscosity of the liquid medium increases, there is a decrease in the frequency of spurious resonances in accordance with the shift of the fundamental resonance and a slight increase in the motional resistance. Even if the increase of the motional resistance is slow for glycerol-water solutions higher than 80%, it is difficult to automatically identify the position of the spurious resonances. The evolution of the electrical parameters of the extended BVD model in liquid media can provide additional information to better describe the interactions occurring at the QCM sensor surface. A deep analysis of the effects of the working medium on the electrical parameters of the extended BVD model is the subject of the next subsection.

### 3.2. Parameters of Interest for Spurious Resonances

The electrical parameters of the extended BVD model provide all the information that can be monitored about the behavior of the QCM sensor regardless of the specifics of an application. In practice, for reasons related to the evolution of QCM instrumentation, the evolution of only a few key parameters of the QCM sensor is retained, usually the evolution of the series resonance frequency and the D-factor. Advanced methods for monitoring key parameters and data processing have been developed for these particular cases [18,19]. The use of a VIA brings all imaginable benefits, with one limitation related to the measurement time, which can be critical in the case of fast interactions occurring at the QCM sensor surface [17]. 

The experimental results are also interpreted in the sense of compatibility with many traditional measurements. In this context, the usefulness of spurious resonances has been highlighted in the transition zone from air to liquid medium but also can be partially demonstrated even in liquid medium. In the case of series frequency monitoring of odd harmonic resonances, their different response provides better experimental support for the interpretation of the phenomena occurring at the QCM sensor surface. Similarly, it is useful to investigate the behavior of spurious resonances in relation to the fundamental resonance in order to reach a conclusion about their usefulness. 

**Figure 9:**
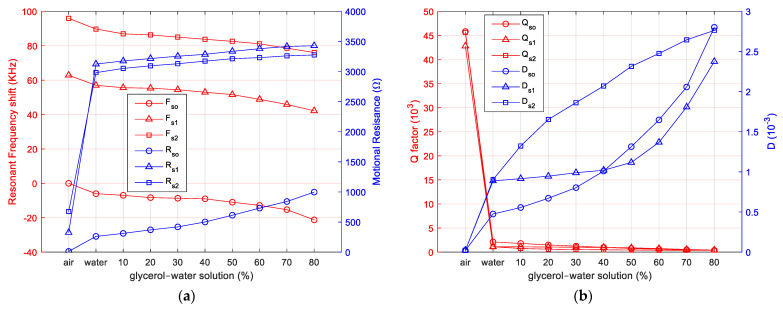
QCM sensor: (**a**) evolution of series resistance and series resonance frequency shift in air, water, and glycerol-water solution; (**b**) evolution of Q-factor and D-factor in air, water, and glycerol-water solution.

This comparative study involves investigating the series resonance frequency shift for spurious resonances relative to the series frequency shift of the fundamental resonance. The evolution of the motional resistance, Q-factor, and D-factor are also parameters of interest in the hypothesis supporting the usefulness of spurious resonances. The evolution of these parameters has been investigated by a set of systematic measurements that have been summarized in Figure 9.

As can be seen in Figure 9a, the series frequency shift of the spurious resonances monitored in this study is identical to the frequency shift of the fundamental series resonance. Also, in Figure 9a, the significant increase of the motional resistance for the spurious resonances at the transition from air to liquid medium (pure water) can be seen. The evolution of the motional resistance for spurious resonances is significantly different from the evolution of the motional resistance of the fundamental series resonance. The motional resistance of spurious resonances increases with increasing viscosity of the liquid medium after a smooth slope. For liquid media, it would seem at first glance that monitoring them is not useful. A different conclusion is drawn from the D-factor analysis shown in Figure 9b. The evolution of the D factor with increasing viscosity is significantly different for spurious resonances, suggesting that it is useful to monitor them in liquid media.

## 4. Discussion

As shown in Figure 9a, tracking spurious series resonance frequencies is not of interest from the perspective of better interpreting the interactions occurring at the QCM sensor electrode surface. The same conclusion is not reached from Figure 9b, where, in the case of the D factor associated with spurious series resonances, the behavior of the QCM sensor with increasing viscosity is significantly different from the evolution of this parameter for the fundamental series resonance. This behavior of spurious series resonances in the case of the D factor makes a crucial contribution to a more detailed analysis of the dissipative phenomena occurring at the QCM sensor surface. 

It is worth mentioning the usefulness of measuring and monitoring the series motional resistances and, implicitly, based on Equation (10), the D factor for spurious series resonances. A distinct favorable situation is created by the significant increase of the motional resistance for spurious resonances at the transition from air to water. In other words, spurious series resonances are much more strongly attenuated than the fundamental series resonance. In this range of dissipation processes between the air and water media, many applications in chemosensors or biosensors, such as VOC sensors [30] or humidity sensors [31], have already been identified. The evolution of the D factor for spurious series resonances with increasing medium viscosity suggests a new candidate for future analysis, providing a better interpretation of the interactions taking place at the QCM sensor surface. 

In this paper, the QCM sensor and its spurious resonances were investigated from the perspective of increasing the viscosity of the working medium. It cannot be argued that the QCM sensor response is identical from the perspective of spurious resonances in any experimentally investigated situation. These differences are not significant as long as the QCM sensors come from the same manufacturer. The dissipation process induced by the glycerol-water solution is considered standard in the literature, so it can be considered that the experimental investigation presented in this study regarding the spurious response of the QCM sensor is representative.

## 5. Conclusions

In this paper, the presence and evolution of spurious resonances as a function of the changes induced by the QCM sensor environment were studied. These investigations revealed a different evolution of the motional resistance of spurious resonances compared to the motional resistance of the fundamental resonance. This situation is very favorable in the case of switching from air to a water medium, in which case the motional resistance of spurious resonances changes significantly, as shown in Table 3. This result confirms the usefulness of monitoring the motional resistance of spurious resonances in specific applications such as VOC sensors, humidity sensors, or dew point sensors. This first experimentally demonstrated conclusion confirms the significant increase in sensitivity of measuring dissipative processes induced in the functionalization layer of a VOC sensor based on the QCM sensor. 

Extensive experimental investigations in the liquid working medium revealed significantly different behavior of the D factor for spurious resonances relative to fundamental resonance. This different evolution may be useful in understanding the dissipative phenomena induced by interactions occurring at the QCM sensor surface. 

## Figures and Tables

**Table 1:** The values of the electrical parameters of the extended BVD model, in air.

*i*	*R_si_* [Ω]	*L_si_* [mH]	*C_si_* [fF]
0	10	8.9475	28.325
1	350	176.74	1.4163
2	700	263.10	0.9441
3	2800	435.65	0.5665
4	3000	519.60	0.4720

**Table 2:** The values of the electrical parameters of the extended BVD model, in water.

*i*	*R_si_*	*L_si_*	*C_si_*
0	250	8.9475	28.325
1	3500	176.74	1.4163
2	4500	263.10	0.9441
3	7500	435.65	0.5665
4	10,000	519.60	0.4720

**Table 3:** Difference between motional resistance *R_s_*_0_ and spurious motional resistance *R_si_*.

	*R_s_*_0_	*R_s_*_1_	*R_s_*_2_
**air**	12.38	325.48	674.77
**water**	261.58	3095.98	3045.2
**Δ** ***R***	249.2	2770.5	2370.43

## Data Availability

Not applicable.
